# The influence of the weight-bearing state on three-dimensional (3D) planning in lower extremity realignment – analysis of novel vs. state-of-the-art planning approaches

**DOI:** 10.1007/s00402-024-05289-3

**Published:** 2024-03-30

**Authors:** Sandro Hodel, Tabitha Arn-Roth, Florian Haug, Fabio Carillo, Lazaros Vlachopoulos, Sandro F. Fucentese, Philipp Fürnstahl

**Affiliations:** 1 Balgrist University Hospital, Department of Orthopedics, University of Zurich, Forchstrasse 340, Zurich, 8008 Switzerland; 2 Balgrist University Hospital, Research in Orthopaedics Computer Science, University of Zurich, Balgrist Forchstrasse 340, Zurich, 8008 Switzerland

**Keywords:** Knee, Osteotomy, Leg alignment, Automatization, Patient-specific instrumentation, Tibia, osteotomy

## Abstract

**Background:**

The use of 3D planning to guide corrective osteotomies of the lower extremity is increasing in clinical practice. The use of computer-tomography (CT) data acquired in supine position neglects the weight-bearing (WB) state and the gold standard in 3D planning involves the manual adaption of the surgical plan after considering the WB state in long-leg radiographs (LLR). However, this process is subjective and dependent on the surgeons experience. A more standardized and automated method could reduce variability and decrease costs.

**Purpose:**

The aim of the study was (1) to compare three different three-dimensional (3D) planning modalities for medial open-wedge high tibial osteotomy (MOWHTO) and (2) to describe the current practice of adapting NWB CT data after considering the WB state in LLR. The purpose of this study is to validate a new, standardized approach to include the WB state into the 3D planning and to compare this method against the current gold standard of 3D planning. Our hypothesis is that the correction is comparable to the gold standard, but shows less variability due compared to the more subjective hybrid approach.

**Methods:**

Three surgical planning modalities were retrospectively analyzed in 43 legs scheduled for MOWHTO between 2015 and 2019. The planning modalities included: (1) 3D hybrid (3D non-weight-bearing (NWB) CT models after manual adaption of the opening angle considering the WB state in LLR, (2) 3D NWB (3D NWB CT models) and (3) 3D WB (2D/3D registration of 3D NWB CT models onto LLR to simulate the WB state). The pre- and postoperative hip-knee-ankle angle (HKA) and the planned opening angle (°) were assessed and differences among modalities reported. The relationship between the reported differences and BMI, preoperative HKA (LLR), medial meniscus extrusion, Outerbridge osteoarthritis grade and joint line convergence angle (JLCA) was analyzed.

**Results:**

The mean (std) planned opening angle of 3D hybrid did not differ between 3D hybrid and 3D WB (0.4 ± 2.1°) (n.s.) but was higher in 3D hybrid compared to 3D NWB (1.1° ± 1.1°) (*p* = 0.039). 3D WB demonstrated increased preoperative varus deformity compared to 3D NWB: 6.7 ± 3.8° vs. 5.6 ± 2.7° (*p* = 0.029). Patients with an increased varus deformity in 3D WB compared to 3D NWB (> 2 °) demonstrated more extensive varus alignment in LLR (*p* = 0.009) and a higher JLCA (*p* = 0.013).

**Conclusion:**

Small intermodal differences between the current practice of the reported 3D hybrid planning modality and a 3D WB approach using a 2D/3D registration algorithm were reported. In contrast, neglecting the WB state underestimates preoperative varus deformity and results in a smaller planned opening angle. This leads to potential under correction in MOWHTO, especially in patients with extensive varus deformities or JLCA.

**Clinical Relevance:**

Incorporating the WB state in 3D planning modalities has the potential to increase accuracy and lead to a more consistent and reliable planning in MOWHTO. The inclusion of the WB state in automatized surgical planning algorithms has the potential to reduce costs and time in the future.

## Introduction

Preoperative planning for lower extremity realignment surgery is widely based on two-dimensional (2D) weight-bearing (WB) long-leg radiographs (LLR), as this modality allows the quantification of the leg axis deviation under WB load [1]. Over the last years, surgical planning modalities based on computed tomography (CT) reconstructed three-dimensional (3D) models have been proposed [2–4]. 3D models can provide the surgeon with a more detailed understanding of the underlying deformity. The use of 3D deformity analysis and surgical planning supports the surgeon in the accurate execution of the pre-defined plan through surgical navigation, such as patient-specific instruments [2]. This allows the concomitant correction in multiple planes and prevents unintentional alterations in sagittal or axial alignment during lower extremity corrections [5–7]. Despite these advancements using 3D planning and navigation, the models rely solely on CT imaging acquired in a non-weight-bearing (NWB) supine position, neglecting the impact of the WB state on the limb (subsequently referred to as 3D NWB) [8, 9]. Therefore, in current practice the surgeon needs to adapt the surgical correction based on 3D NWB CT models after considering the extent of preoperative deformity under WB conditions in LLR (subsequently referred to as 3D hybrid). However, this decision is dependent on the individual surgeon’s experience and therefore the planning modality lacks standardization and validation, which leads to undesired variability, limited reproducibility and comparability in the literature up to date. Additionally, manual planning is associated with higher costs and time, which could be addressed with automated planning algorithms in the future. D.

Previous studies have investigated the use of 3D/2D registration algorithms to transform CT imaging data onto 2D x-ray modalities for various clinical use cases [10]. For corrective osteotomies of the lower extremities, Roth. et al. proposed a registration algorithm to transform the 3D NWB CT model into the WB state [11]. The 3D/2D registration of 3D NWB CT models onto biplanar standing LLR enables 3D surgical planning under consideration of the WB state (subsequently referred to as 3D WB).

However, the use of a 3D WB modality to plan medial open-wedge high tibial osteotomies (MOWHTO) has not been studied yet and differences to current planning approaches (3D NWB and 3D hybrid) are not validated. Therefore, the aim of this study is to validate a new, standardized approach to include the WB state into the 3D planning and to compare this method against the current gold standard of 3D planning. Our hypothesis is that the correction is comparable to the gold standard, but shows less variability due compared to the more subjective hybrid approach.

## Methods

### Study cohort

After ethical approval (ID 2017 ? 01616), 59 consecutive legs identified from a previous study that underwent biplanar LLR and a standardized lower extremity CT protocol at our institution from 2015 to 2019 were screened [11]. Inclusion criteria comprised a complete pre- and postoperative biplanar LLR, a standardized CT protocol (including the hip, knee and ankle joints) and complete documentation of the preoperative surgical planning.

After excluding patients with insufficient preoperative documentation of the surgical planning or deformity analysis (*n* = 9), torsional realignment (*n* = 2), extensive flexion position in LLR (> 20°) (*n* = 3), a shaft correction (*n* = 1), and a planned varization (*n* = 1), 43 legs (41 patients) planned for an MOWHTO due to medial knee degeneration or focal chondral defects that underwent cartilage surgery were available for definitive analysis. Demographic characteristics are summarized in Table 1.

**Table 1:** Demographics, frontal leg alignment, and medial compartment degenerative findings

	43 legs (100%)
Age (years)	43.6 ± 10.1
Male gender	34 (79.1)
BMI (kg/m^2^) Height (cm) Weight (kg)	29.8 ± 4.9 173.4 ± 7.4 89.5 ± 13.9
HKA (°) *	6.9 ± 3.2
mMPTA (°) *	85.5 ± 2.7
mLDFA (°) *	89.6 ± 1.9
JLCA (°) **	3.4 ± 1.9
Outerbridge grade ***	
I II III IV	4 (9.3) 7 (16.3) 13 (30.2) 16 (44.2)
Medial meniscus extrusion (mm)	4.8 ± 1.1

BMI: Body-mass index. HKA: Hip-knee-ankle angle (+ varus, - valgus). mMPTA mechanical medial proximal tibial angle. mLDFA: mechanical lateral distal femoral angle. JLCA: Joint line convergence angle (+ lateral opening, - medial opening). Data presented as n (%) or mean ± standard deviation if not stated otherwise. * Measured in long leg radiograph. ** Measured in standing x-ray. *** Three patients did not undergo preoperative magnetic-resonance imaging

We sought to analyze three different preoperative planning methods: (1) the current standard planning modality at our institution 3D NWB CT model-based planning which was manually adapted based on standing LLR (referred to as 3D hybrid), (2) 3D NWB CT model-based planning (referred to as 3D NWB), and 3)3D NWB CT model-based planning after a 3D/2D registration onto standing LLR to simulate the WB state (referred to as 3D WB). The three modalities are explained in detail in the paragraphs hereafter. The preoperative planning was retrospectively reviewed for each patient and modality (3D hybrid, 3D NWB, 3D WB).

### Radiographic assessment

Radiographic assessment of leg alignment included biplanar standing LLR (EOS Imaging, Paris, France). The frontal LLR was imported to mediCAD® software (Hectec GmbH, Altdorf, Germany) and calibrated. After manual definition of landmarks (center of the femoral head, apex of the greater trochanter, femoral and tibial joint line, medial and lateral border of the femoral condyles and tibial plateau, medial and lateral border of the talus, and the joint line of the talus), automatic deformity analysis is performed by the software according to Paley et al. [12]. Preoperative deformity analysis included HKA (°) (+ varus, - valgus), mechanical medial proximal tibia angle (°) (mMPTA) and mechanical lateral distal femoral angle (°) (mLDFA). Joint line convergence angle (°) (JLCA) (+ lateral opening, - medial opening) was measured in standing x-ray. Additionally, medial meniscus extrusion (mm) [13] and Outerbridge grade of the medial compartment [14] were assessed in preoperative magnetic-resonance imaging(MRI) if preoperative MRI was available by a blinded investigator who was not involved in clinical care of the patients. Preoperative leg alignment and MRI findings are summarized in Table 1.

### Computer-tomography protocol and three-dimensional models

All patients underwent preoperative supine CT of the lower extremity according to a standardized protocol (MyOsteotomy, Medacta, Castel San Pietro, Switzerland) comprising all anatomical structures of interest: hip center, proximal femur, knee center with distal femur and proximal tibia, ankle joint center with distal tibia, distal fibula and talus. 3D surface models of the lower extremities were created using global threshold segmentation and region growing with MIMICS software (MIMICS, Materialize, Belgium) and imported into the in-house developed surgical planning software (Xxxxxxxxxx). A 3D coordinate system was defined according to the International Society of Biomechanics (ISB) [15]. The hip center was defined as the center of a sphere, fitted to the femoral head. The knee center was defined as the midpoint between the intercondylar eminences on the tibial plateau and the ankle center was defined as the center of the distal articular surfaces of the tibia and fibula [8].

### Planning modalities

For all planning modalities the preoperative HKA (referred to as HKA *pre*) (°), planned postoperative HKA (referred to as HKA *post*) (°) and the planned opening angle (°) were measured. The extent of correction to shift the weight-bearing line (WBL) 50–65% on the tibial plateau was defined on an individual basis, considering the indication for realignment, concomitant lateral and patellofemoral degeneration and the joint line obliquity (mMPTA < = 93°) as described by Feucht et al. [16]. The aimed postoperative correction (% of WBL on tibial plateau) was identical in all planning modalities.

### 3D hybrid

**Fig. 1:**
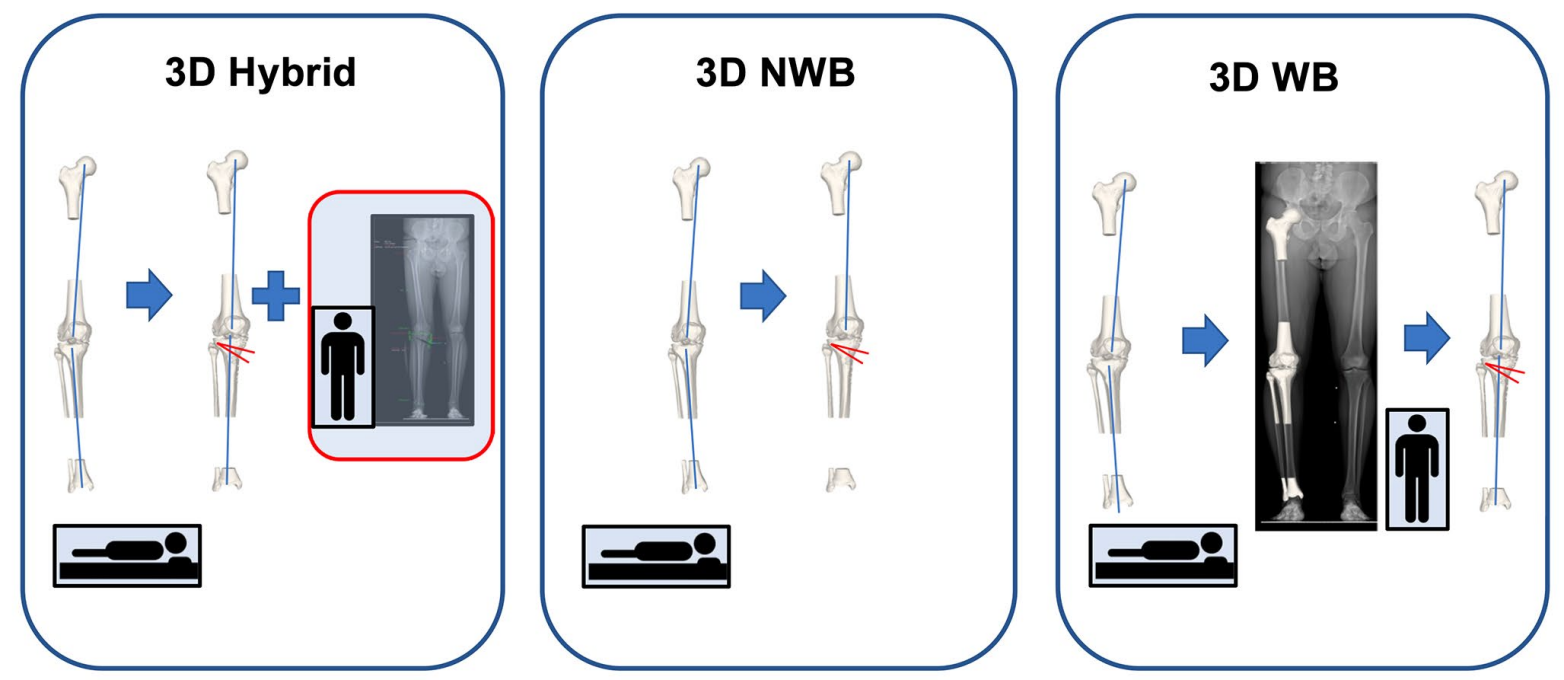
Overview of planning modalities. The planned correction of the mechanical leg axis was analyzed in 3D hybrid (left), 3D non-weight-bearing (NWB) (middle) and 3D weight-bearing (WB) modality (right) for each patient. Left: 3D hybrid is based on NWB 3D CT models and adapted by the surgeon after consideration of WB information from the long-leg-radiograph (LLR) (red box). Middle: 3D NWB relies solely on NWB information from supine acquired 3D CT models. Right: 3D WB allowed the analysis of 3D models in the WB-state after 3D/2D registration of NWB CT models onto standing LLR. The mannikin indicates the lying or standing position during image acquisition for each modality

3D planning for lower extremity realignment surgery represents the state-of-the art method at our institution. After preliminary planning of realignment surgery in 3D NWB CT [2], the definitive planned opening angle is manually adapted by the treating surgeon after considering the extent of deformity in standing LLR and is therefore referred to as 3D hybrid. Increasing the initially suggested opening angle of the 3D NWB plan was considered in patients with increased HKA in LLR compared to the 3D NWB model, to adjust for the missing WB state in the 3D models. For each 3D model, HKA pre and HKA post were measured as the projected 2D angle in the frontal plane between a line defined by hip and knee center, and a line defined by knee and ankle center. The medial opening angle of the tibia was calculated around a single 3D axis of rotation. This 3D planning modality has been previously described and demonstrated excellent reliability [2, 5, 17] (Fig. 1, left).

### 3D non-weight-bearing (NWB)

3D NWB planning relies solely on the CT models obtained in a supine position without additional information of the WB state. The protocol included exactly the same CT scan, creation of 3D models, definition of joint centers and measurements as described above for the 3D hybrid method. However, the deformity analysis and correction were based on supine imaging only and the WB state was not considered (Fig. 1, middle).

### 3D (weight-bearing (WB)

The consideration of the WB state in 3D planning represents a novel planning modality [11]. An intensity-based algorithm was used to register NWB CT scans onto standing LLR to transform the patient-specific 3D models into the WB state. After annotation of the corresponding anatomical landmarks (ankle center, knee center, hip center) in EOS and CT NWB [11], the registration algorithm detects the optimal position by comparing intensity values between the LLR and digitally reconstructed radiographs (DRR) generated from CT using the same geometry as the EOS system [18]. The registration was executed using ImFusion Suite software (ImFusion GmbH, Munich, Germany). Once registration to the WB state was completed, the measurement of HKA pre and HKA post was performed as described for the 3D hybrid method (Fig. 1, right).

Mean differences between the opening angle of 3D hybrid, 3D NWB and 3D WB were calculated. Additionally, the influence of body-mass index (BMI), preoperative HKA in LLR, medial compartment osteoarthritis grade (Outerbridge) [14], medial meniscus extrusion and JLCA on the reported differences were investigated.

### Statistical analysis

The normal distribution of the data was tested with Shapiro-Wilk’s test. Data are reported as mean ± standard deviation (std) or counts (percentages).

Differences between planned correction of HKA among the three planning modalities were tested with Friedman’s test and post-hoc tests were Bonferroni corrected. The planned opening angle for the three methods were analyzed using equivalence testing after calculating tge effect sizes (Cohen’s D) assuming a clinically meaningful border that demonstrates equivalence with a 90% CI of -0.5 to 0.5°. The relationship between the adaption of the opening angle, BMI, preoperative HKA, medial compartment osteoarthritis grade, medial meniscus extrusion, JLCA and the reported differences of 3D NWB and 3D WB were analyzed using Spearman’s rank correlation *(r*_*s*_). Characteristics between a subgroup of patients with > 2° difference between preoperative varus alignment in 3D WB compared to 3D NWB and a subgroup with < 2° difference were reported (metric: student’s t-test, categoric: Chi-Square test). Significance was set at the level of *p* < 0.05. Data were analyzed with SPSS version 26 (SPSS Inc, Chicago, IL, USA).

## Results

**Fig. 2:**
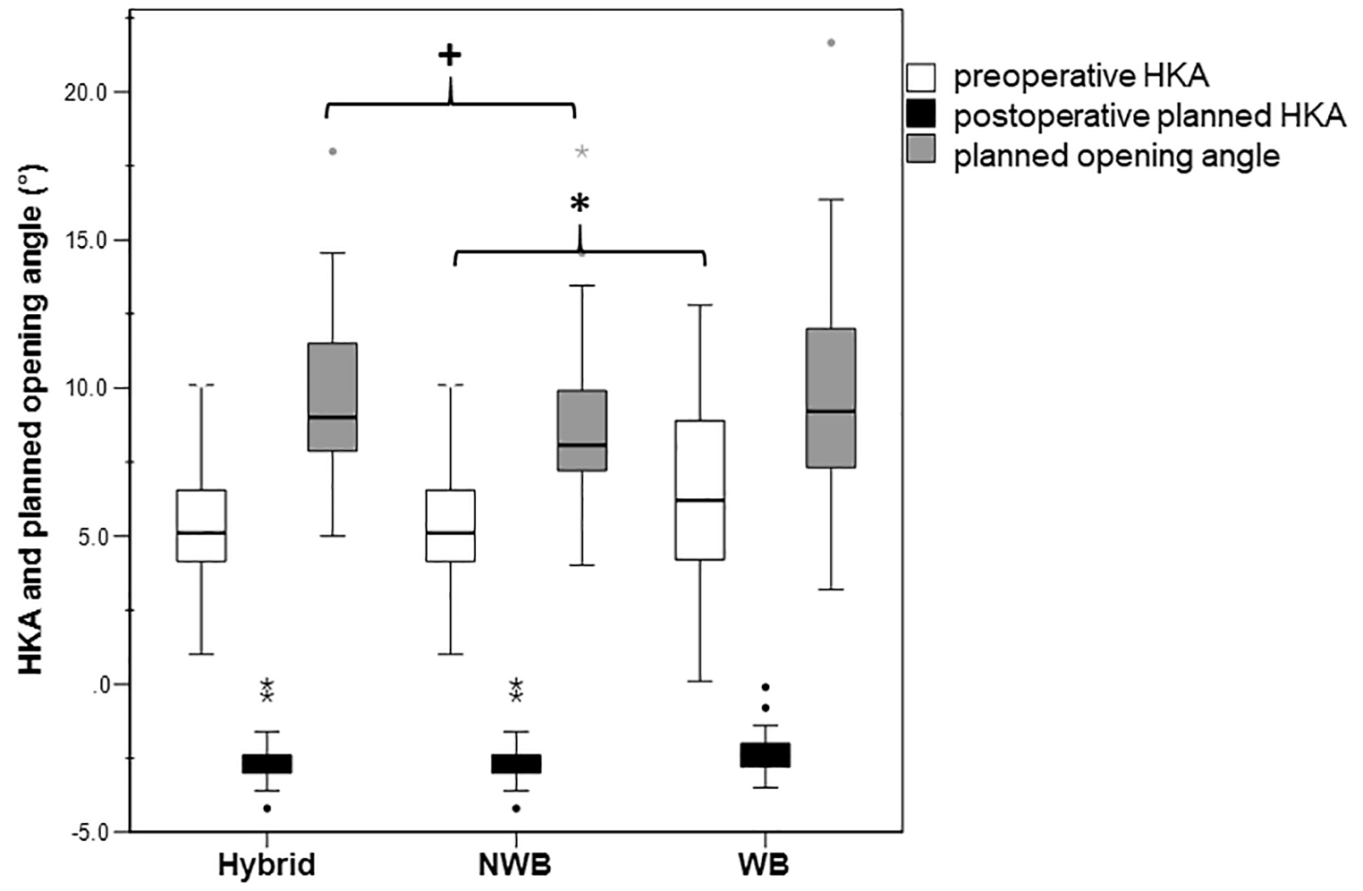
Planned correction of hip-knee ankle angle (HKA) among modalities. Boxplots depicts median (line), IQR (box), minimum and maximum (whisker), and outliers (stars) among planning modalities Preoperative deformity of hip-knee-ankle angle (HKA) was significantly smaller in 3D NWB compared to 3D WB (*p* = 0.029, Bonferroni corrected, depicted by *). The opening angle was significantly higher in the 3D hybrid method compared to the 3D NWB (*p* = 0.039, Bonferroni corrected, depicted by +). The remaining measurements did not differ among the three planning modalities (n.s.)

The mean (std) planned opening angle did not differ between 3D hybrid and 3D WB (0.4 ± 2.1°) (n.s.) but was higher in 3D hybrid compared to 3D NWB (1.1° ± 1.1°) (*p* = 0.039) (Fig. 2). The 3D hybrid and 3D WB demonstrated equivalence (Cohen’s D CI: -0.25 to 0.46), whereas the 3D hybrid and 3D NWB were non-equivalent (Cohen’s D CI: -0.61 to 0.09). The detailed results of all planning modalities are listed in Table 2. The adapted correction based on LLR in the 3D hybrid method correlated with the difference between preoperative HKA of CT WB and NWB *r*_*s*_ =0.54 (*p* = 0.001).

**Table 2:** Differences among planning modalities

	3D hybrid	3D NWB	3D WB	*p*-value (post-hoc)
**HKA (°)**				
Pre	5.6 ± 2.7^*A*^	5.6 ± 2.7^*B*^	6.7 ± 3.8^*A, B*^	*p*=0.029^*A, B*^
Post	-2.6 ± 0.7	-2.6 ± 0.7	-2.4 ± 0.7	n.s.
Opening angle	9.4 ± 2.8 ^*C*^	8.7 ± 2.8 ^*C*^	9.8 ± 3.9	*p*=0.039^*C*^
**Difference in (°) compared to 3D hybrid**				
Pre	NA	NA	1.1 ± 2.2	NA
Post	NA	NA	0.3 ± 0.7	
Opening angle	NA	-1.1 ± 1.1	0.4 ± 2.1	

HKA: hip-knee-ankle angle (+ varus, - valgus). NWB: non-weight-bearing. NA: Not applicable. Data presented as mean ± standard deviation. Significant values marked **bold**. Paired superscript letters depict post-hoc significance between groups (Bonferroni corrected)

**Table 3:** Characteristics of patients with increased varus alignment under weight-bearing load

	> 2° varus alignment in 3D WB vs. 3D NWB (*n* = 15)	< 2° varus alignment in 3D WB vs. 3D NWB (*n* = 28)	*p*-value
**BMI (kg/m** ^**2**^ **)**	31.0 ± 4.0	29.0 ± 5.2	n.s.
Preoperative HKA (°) *	8.7 ± 3.6	5.9 ± 2.8	**0.009**
**Medial compartment** Outerbridge grade **			
**I** **II** **III** **IV**	2 (14.3) 2 (14.3) 3 (21.4) 7 (50.0)	2 (7.7) 6 (23.1) 10 (38.5) 8 (30.8)	n.s.
**Medial meniscus extrusion (mm)**	4.1 ± 1.4	3.8 ± 1.2	n.s.
JLCA (°)***	4.4 ± 2.0	3.0 ± 1.7	**0.013**

BMI: Body-mass index. HKA: Hip-knee-ankle angle (+ varus, - valgus). JLCA: Joint line convergence angle (+ lateral opening, - medial opening). Data presented as n (%) or mean ± standard deviation if not stated otherwise. * Measured in long leg radiograph. ** Three patients did not undergo preoperative magnetic-resonance imaging. *** Measured in standing x-ray

## Discussion

The most important finding of this study is that the described 3D hybrid and 3D WB modality for planning lower extremity realignment demonstrated small intermodal differences, while the 3D NWB approach significantly underestimated the preoperative varus deformity and therefore resulted in a decreased planned opening angle in MOWHTO. The simulation of the WB state with the described 3D/2D registration algorithm represents a valid alternative to the current standard 3D hybrid planning approach. While the differences for 2D planning methods for HTO have been studied before [19], this is the first study to report the differences among state-of-the art 3D planning methods for corrective osteotomies with and without consideration of the WB state.

Preoperative planning of MOWHTO allows the identification of the deformity site and subsequent accurate lower extremity realignment [20]. Accurate surgical planning and execution are crucial to avoid under- and over correction and adequately unload the degenerative medial compartment [21, 22] without creating an oblique joint line [23, 24]. Therefore, currently conventional planning still relies on standing 2D LLR, which allows the consideration of the WB state. While the adaption towards 3D planning modalities has gained attention, including the WB state has only been proposed recently [11]. This study is the first to report the differences between different 3D modalities (WB vs. NWB) in surgical planning of lower extremity realignment. The analyzed surgical planning in 3D WB allows a reliable planning with a mean difference of 0.4° compared to the current standard planning using the 3D hybrid method. Including the WB information in an automatized planning algorithm, without the need of a cumbersome method to account for the missing WB information in 3D CT models acquired in supine position could potentially reduce cost, time and undesired surgeon-dependent variability in the future [25]. 

The extent of preoperative varus deformity and increasing JLCA were identified as contributing factors that explain the difference between preoperative HKA of 3D NWB and 3D WB. Patients with increasing varus deformity under WB load demonstrated increasing meniscus extrusion and medial cartilage degeneration, however, not significant in our cohort. An increase of preoperative varus deformity shifts the load to the medial side of the knee and may explain the differences of preoperative HKA between 3D NWB and 3D WB in the presence of medial compartment degeneration and joint space narrowing. The combination of cartilage degeneration, meniscus extrusion and ligament laxity on the lateral side leads to closing of the medial articular joint space under load and is in line with previous findings [26–29]. However, several other factors contributing to the reported differences between 3D WB and 3D NWB need to be considered. First, LLR measurements are dependent on patient positioning, knee flexion and beam height [30] and therefore influence the 3D/2D registration of the reported 3D WB protocol. Second, the 3D WB modality inherently includes the reported translational and rotational registration errors [11].

A limitation of this study is that the surgical planning was retrospectively reviewed in a single-center cohort. Therefore, the decision to adapt the 3D hybrid method by the treating surgeon is based on multiple factors and only the main ones could be described in the current study. However, this represents current clinical practice, especially as the optimal amount of leg axis correction remains controversial [16, 31]. Additionally, the analyzed cohort included patients planned for MOWHTO with varus deformity only. Therefore, these findings need to be validated for femoral-sided and valgus corrections in further studies. Future research should focus on automatic inclusion of the WB state in 3D planning to minimize undesired user-dependent heterogeneity in surgical planning of lower extremity realignment.

## Conclusion

Small intermodal differences between the current practice of the reported 3D hybrid planning modality and a 3D WB approach using a 2D/3D registration algorithm were reported. In contrast, neglecting the WB state underestimates preoperative varus deformity and results in a smaller planned opening angle. This leads to potential under correction in MOWHTO, especially in patients with extensive varus deformities or JLCA.

### Clinical relevance

Incorporating the WB state in 3D planning modalities has the potential to increase accuracy and lead to a more consistent and reliable planning in MOWHTO. The inclusion of the WB state in automatized surgical planning algorithms has the potential to reduce costs and time in the future.
